# An oral health optimized diet can reduce gingival and periodontal inflammation in humans - a randomized controlled pilot study

**DOI:** 10.1186/s12903-016-0257-1

**Published:** 2016-07-26

**Authors:** J. P. Woelber, K. Bremer, K. Vach, D. König, E. Hellwig, P. Ratka-Krüger, A. Al-Ahmad, C. Tennert

**Affiliations:** 1 Department of Operative Dentistry and Periodontology, Center for Dental Medicine, Medical Center – University of Freiburg, Hugstetter Str. 55, Freiburg, Germany; 2 Department of Medical Biometry and Statistics, Medical Center – University of Freiburg, Freiburg, Germany; 3 Institute of Sports and Sports Science, Medical Center – University of Freiburg, Freiburg, Germany

**Keywords:** Periodontal diseases, Gingivitis, Diet, Food and nutrition, Carbohydrates, Fatty acids, Omega 3, vitamins

## Abstract

**Background:**

The aim of this pilot study was to investigate the effects of four weeks of an oral health optimized diet on periodontal clinical parameters in a randomized controlled trial.

**Methods:**

The experimental group (*n* = 10) had to change to a diet low in carbohydrates, rich in Omega-3 fatty acids, and rich in vitamins C and D, antioxidants and fiber for four weeks. Participants of the control group (*n* = 5) did not change their dietary behavior. Plaque index, gingival bleeding, probing depths, and bleeding upon probing were assessed by a dentist with a pressure-sensitive periodontal probe. Measurements were performed after one and two weeks without a dietary change (baseline), followed by a two week transitional period, and finally performed weekly for four weeks.

**Results:**

Despite constant plaque values in both groups, all inflammatory parameters decreased in the experimental group to approximately half that of the baseline values (GI: 1.10 ± 0.51 to 0.54 ± 0.30; BOP: 53.57 to 24.17 %; PISA: 638 mm^2^ to 284 mm^2^). This reduction was significantly different compared to that of the control group.

**Conclusion:**

A diet low in carbohydrates, rich in Omega-3 fatty acids, rich in vitamins C and D, and rich in fibers can significantly reduce gingival and periodontal inflammation.

**Trial registration:**

German Clinical Trials Register; https://www.germanctr.de (DRKS00006301). Registered on 2015-02-21.

**Electronic supplementary material:**

The online version of this article (doi:10.1186/s12903-016-0257-1) contains supplementary material, which is available to authorized users.

## Background

Periodontal disease is a global burden affecting about 743 million people worldwide and is considered to be a primary cause of tooth loss at advanced age [1, 2]. Gingivitis is a prerequisite for the development of periodontal disease, and also correlates to long-term tooth loss [3]. A landmark study in 1965 by Löe and coworkers demonstrated the influence of dental plaque as an etiological factor for gingival inflammation by showing increased gingival inflammation when participants discontinued oral hygiene procedures [4]. However, Brecx et al. [5] showed that individuals reacted differently regarding their inflammatory response to plaque accumulation, with some individuals showing only a mild expression of gingival inflammation. A study by Baumgartner et al. [6] looking at participants during a stone-age experiment showed pronounced reductions of gingival and periodontal inflammation, even though oral hygiene was not performed at all. The authors concluded that the experimental gingivitis protocol is not applicable if the diet does not include refined carbohydrates. Thus, diet seems to have a profound impact on the gingival and periodontal inflammatory reaction. Examining the literature, several dietary recommendations for benefiting the health of periodontal tissues can be found, such as a reduction in carbohydrates, and an additional intake of Omega-3 fatty acids, vitamin C, vitamin D, antioxidants and fiber [7–13].

Most importantly, the excessive intake of carbohydrates seems to promote dysbiosis and chronic inflammatory diseases [8, 14]. The clinical reduction of carbohydrate intake seems to reduce gingival inflammation [7]. In-vitro studies showed that high levels of glucose promote apoptosis and inhibit proliferation of periodontal ligament cells [15, 16].

Secondly, an imbalance between Omega-6 and Omega-3 fatty acids seems to foster inflammation. Reports indicate that the Omega-6 to Omega-3 ratio changed from 1:1 in a hunter-gatherer diet to 15:1 in a Western diet, accompanied by higher blood levels of several cytokines [10]. Interestingly, there is growing evidence that Omega-3 fatty acids lead to resolution of inflammatory processes [9]. The resolution of inflammation was also shown for periodontal tissues in vitro [17], while positive therapeutic effects were seen in two clinical studies in the field of periodontal therapy [18, 19].

Furthermore, the intake of vitamins C and D seem to play an important role in gingival and periodontal inflammation. Several studies showed the positive impact of vitamin D on periodontal tissues both in clinical and in-vitro studies [11, 20, 21]. Vitamin C has been described as an important vitamin for periodontal health, both in clinical and in-vitro studies for some time. The absence of vitamin C causes scurvy, which is accompanied by massive periodontal bone loss [22–25].

Last but not least, the role of dietary antioxidants seems to be important for several processes regarding an adequate systemic reaction to oxidative stress. Both clinical and *in-vitro* studies showed positive effects on periodontal tissues [11, 12, 26–28].

Despite these very promising findings, there is a substantial lack of dietary-interventional studies in controlled randomized settings [11]. Thus, the aim of this pilot study was to evaluate an oral health optimized diet low in carbohydrates, and rich in Omega 3-fatty acids, vitamins C and D, antioxidants and rich in fiber in a controlled, randomized study.

## Methods

### Ethics and trial registration

Prior to patient recruitment the study was approved by the University of Freiburg Ethics committee (Reference number 338/14) and registered in an international clinical trial register (German Clinical Trials Register; DRKS00006301; https://www.germanctr.de/).

### Inclusion criteria

- age ≥ 18 years
- patients with gingivitis (GI > 0,5)
- a diet based primarily on carbohydrates [29]

### Exclusion criteria

- smoking
- infectious or life-threatening diseases
- intake of antibiotics within 3 months before the start of or during the study period.
- drugs influencing gingival inflammation or bleeding (e.g. anticoagulants, cortisone)
- carbohydrate- or insulin-related diseases (e.g. diabetes)
- pregnancy or breastfeeding

### Patient recruitment

Patients were recruited in the Department of Operative Dentistry and Periodontology, Medical Center - University of Freiburg, Germany. Freiburg is located in the south-west of Germany with approximately 220.000 inhabitants. Patients were informed about the study and in case of gingival inflammation assessed using the gingival index by Löe & Silness [30] asked for participation. At this stage no further periodontal assessment was performed. Exclusion criteria were checked (by CT) including the prerequisite of a diet mainly based on carbohydrates by means of a verbal dietary anamnesis. After receiving written consent, participants were randomly assigned to the experimental group or the control group following a web-generated randomization list.

### Dietary recommendations

Dietary recommendations were based on the current literature with regard to diet and general inflammation [9, 31], and gingival / periodontal inflammation [7, 11]. The dietary pattern was finally checked by a specialist in internal medicine, nutrition and diabetology (DK).

Dietary pattern in the experimental group included the following elements:

- Reduction of the intake of carbohydrates as far as possible to a level <130 g/d, which can be considered as a low-carb diet [29]. This included a restriction in the amount of fructose, disaccharides, sweetened beverages and meals, flour containing foods, rice and potatoes as far as possible. There were no restrictions regarding fruits and vegetables (polysaccharides) as long as the total amount of carbohydrates was considered.
- Daily intake of Omega-3 fatty acids (such as fish oil capsules, a portion of sea fish, two spoons of flaxseed oil etc.), a restriction in the amount of trans-fatty acids as far as possible (such as fried meals, crisps, donuts, croissants etc.) and a reduction in Omega-6 fatty acids as far as possible (such as safflower oil, grape seed oil, sunflower oil, margarine, sesame oil, corn oil etc.).
- Daily intake of a source of vitamin C (like two kiwis, one orange, one bell pepper etc.)
- Daily intake of a source of vitamin D (15 min unprotected in the sun, supplementation with 500 international units (12.5 μg), 300 g Avocado, etc).
- Daily intake of antioxidants (such as a handful of berries, cup of green tea, coffee etc.)
- Daily intake of fiber (vegetables and fruits).

Dietary recommendations were delivered verbally (30 min) and by handing out an information brochure containing an additional list of restricted and recommended foods and meals. After one week, participants were asked about their experiences and possible problems. When more information was needed, participants had the chance to contact two of the authors at any time during the study (JPW, CT). All participants had to fill out a daily food diary (Additional file 1) throughout the study duration, and to return the diaries at the current appointment..

### Clinical measurements and procedures

Clinical measurements were performed by a dentist blinded to the kind and group of intervention (KB) and included assessment of a gingival index (GI by Löe & Silness [30], plaque index (PI by Silness & Löe [32], a full-mouth periodontal examination including pocket probing depths (PD), bleeding on probing (BOP), and recessions at six sites of the teeth respectively. The periodontal status was assessed using a pressure-sensitive probe (DB764R, Aesculap AG, Tuttlingen, Germany). After assessing the full-mouth periodontal status the first quadrant was examined twice in order to calculate the intra-rater variance. The data were saved digitally via specialized periodontal software (Parostatus.de, Parostatus.de GmbH, Berlin, Germany), which was also used to calculate the total periodontal inflamed surface area (PISA) according to Nesse et al. [33].

For both groups the baseline was assessed in two appointments after one and two weeks. At the start of the first week all participants were instructed to stop all interdental hygiene procedures for the next eight weeks. The first baseline measurement included oral hygiene indices (GI, PI). At the second appointment oral hygiene indices were assessed once again and the periodontal status (PD, BOP, recessions) was evaluated. Participants were encouraged not to change their physical activity and oral hygiene behavior during the entire study, and to stop any interdental cleaning. In case of detected periodontal lesions participants were informed about their disease and scheduled for periodontal treatment after the study.

After the final baseline measurement, patients of the experimental group had an appointment for the dietary instructions with one of the authors (JPW, CT). The following two weeks were seen as a transition time for the patients in order to identify possible problems in changing their dietary behavior to the new diet. After these two transition weeks, patients of the experimental group were encouraged to follow the oral health optimized diet patterns for four weeks to the best of their ability. This observation period was based on the study by Baumgartner et al. [6]. In this period, GI and PI were assessed weekly. At the end of the four weeks total periodontal status was again assessed. As a reward for participating in the study, the patients were given an electric toothbrush with a value of about 70 Euro.

Patients in the control group were evaluated using the same schedule, but were encouraged to continue their dietary habits.

All participants were measured in height and weight in order to calculate the Body Mass Index (BMI). Furthermore, patients were encouraged not to talk to the rating dentist (KB) about study related content.

The food diaries were analyzed with regard to the degree of the participants’ compliance in fulfilling the recommended diet.

### Statistical procedures

Sample size calculation was based on the Baumgartner et al. [6] study with a reduction in BOP from 34.8 (±24.3) to 12.6 (±10). Accordingly, with a power = 80 % and alpha = 5 %, *n* = 12 participants would be needed. Due to the assumption of correlated participants (population of family members) in the Baumgartner study, *n* = 10 participants were aimed for current sample size in an independent population. The control group of *n* = 5 participants was not determined by sample size calculation. The study was considered as a pilot study. Mean BOP was considered as the primary outcome variable, GI, PI, PISA as secondary outcome variables.

Randomization was performed by one of the authors (JPW) using a web-based randomizer (www.random.org, Randomness and Integrity Services Ltd., Dublin, Ireland) with a presetting of five controls and ten experimentals.

The descriptive analysis included median, mean, standard deviation and minimum and maximum. For testing the differences between both groups, a mixed linear regression analysis was performed. Multiple testing was corrected using the Scheffe method. Intra-rater variance was analyzed by calculating the intraclass correlation coefficient (ICC) for the rating dentist (KB). All analyses were performed with a statistical software (STATA 13.1, StataCorp, Texas, USA).

## Results

In total, 16 patients with gingivitis were recruited. In the experimental group, one participant dropped out of the study due to a lack of time for study participation.

The experimental group consisted of 10 participants with 6 women and 4 men. The mean age in this group was 34.4 ± 14.1 years, ranging from 23 to 70 years. The mean BMI was 24.53 ± 2.53 kg/m^2^.

The control group consisted of 5 participants with 3 women and 2 men. The mean age in this group was 34.0 ± 16.5 years, ranging from 24 to 63 years. The mean BMI was 21.98 ± 3.24 kg/m^2^.

**Fig. 1:**
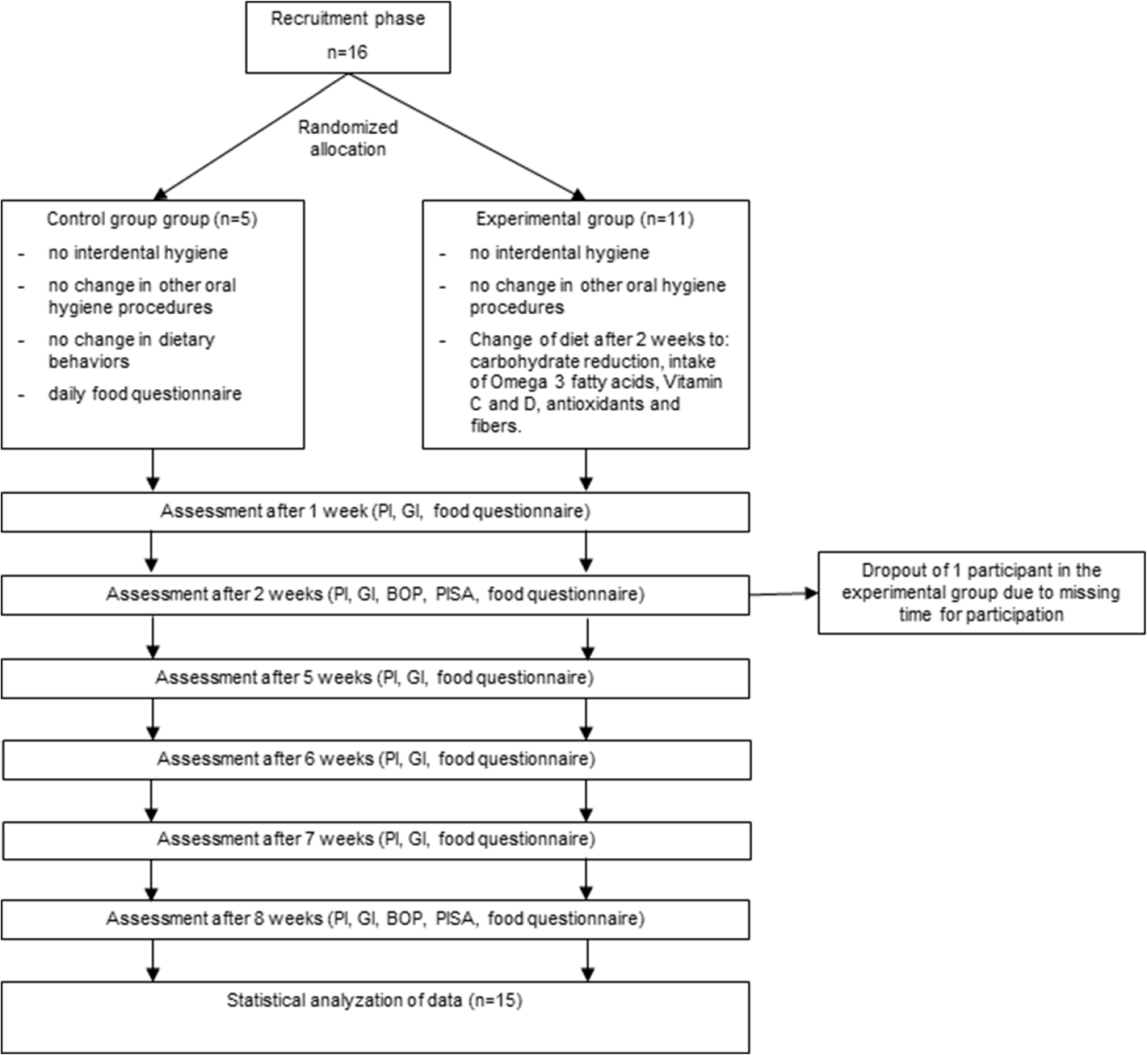
Flow chart of procedures

Clinical data regarding PI, GI, PD, CAL, BOP and PISA are presented in Table 1. A flow chart of the procedures is presented in Fig. 1. In the experimental group, one patient suffered from mild periodontitis and one patient from moderate periodontitis [34]. In the control group, two patients suffered from mild periodontitis.

**Table 1:** Clinical data of both groups for all weeks

Factor	Group	Week 1	Week 2	Week 5	Week 6	Week 7	Week 8
PI	Experimental	0.77 (0.52)	0.88 (0.48)	0.89 (0.51)	0.85 (0.46)	0.80 (0.52)	0.84 (0.47)
	Control	0.75 (0.63)	0.81 (0.46)	0.94 (0.51)	0.84 (0.53)	0.88 (0.56)	0.97 (0.70)
GI	Experimental	1.10 (0.51)	1.20 (0.30)	0.80 (0.42)	0.62 (0.37)	0.54 (0.39)	0.54 (0.30)
	Control	1.01 (0.14)	1.04 (0.17)	1.30 (0.17)	1.38 (0.15)	1.30 (0.19)	1.22 (0.17)
PD	Experimental	-	2.19 (0.34)	-	-	-	2.11 (0.35)
	Control	-	2.31 (0.43)	-	-	-	2.52 (0.40)
CAL	Experimental	-	2.31 (0.52)	-	-	-	2.22 (0.47)
	Control	-	2.53 (0.90)	-	-	-	2.76 (0.88)
BOP (%)	Experimental	-	53.57 (18.65)	-	-	-	24.17 (11.57)
	Control	-	46.46 (15.61)	-	-	-	64.06 (11.27)
PISA (mm^2^)	Experimental	-	638.88 (305.41)	-	-	-	284.83 (174.14)
	Control	-	662.24 (420.05)	-	-	-	963.24 (373.78)

*PI* Clinical results regarding plaque index, *GI* gingival index, *PD* pocket depth, *CAL* clinical attachment level, *BOP* bleeding on probing, and *PISA* periodontal inflamed surface areas with mean values with the standard deviations in parentheses

Regression analysis adjusted for age, gender and BMI revealed no significant differences between the groups regarding PI (*p* = 0.084), but significant differences regarding GI (*p* < 0.001), BOP (*p* = 0.012), and PISA (*p* < 0.001).

Table 2 presents the degree of compliance regarding dietary recommendations.

**Table 2:** Degree of compliance to dietary recommendations

Factor	Group	Week 1	Week 2	Week 5	Week 6	Week 7	Week 8
Vitamin C	Experimental	0.46 (0.29)	0.49 (0.25)	0.70 (0.37)	0.80 (0.20)	0.76 (0.30)	0.79 (0.28)
	Control	0.56 (0.20)	0.56 (0.25)	0.56 (0.32)	0.50 (0.34)	0.40 (0.31)	0.46 (0.22)
Omega-3 fatty acids	Experimental	0.04 (0.10)	0.07 (0.11)	0.82 (0.33)	0.82 (0.36)	0.87 (0.30)	0.83 (0.33)
	Control	0.24 (0.43)	0.24 (0.43)	0.24 (0.43)	0.24 (0.43)	0.22 (0.44)	0.22 (0.44)
Fibers	Experimental	0.40 (0.29)	0.38 (0.27)	0.90 (0.17)	0.88 (0.16)	0.94 (0.14)	0.93 (0.15)
	Control	0.50 (0.19)	0.64 (0.30)	0.44 (0.27)	0.48 (0.39)	0.48 (0.38)	0.48 (0.38)
Vitamin D	Experimental	0.13 (0.31)	0.11 (0.31)	0.41 (0.45)	0.42 (0.48)	0.43 (0.47)	0.47 (0.46)
	Control	0.24 (0.43)	0.22 (0.44)	0.22 (0.44)	0.22 (0.44)	0.32 (0.46)	0.22 (0.44)
Antioxidants	Experimental	0.70 (0.42)	0.76 (0.41)	0.87 (0.29)	0.77 (0.42)	0.93 (0.22)	0.89 (0.31)
	Control	0.60 (0.55)	0.48 (0.38)	0.56 (0.45)	0.62 (0.41)	0.58 (0.36)	0.54 (0.39)
Carbohydrate reduction	Experimental	0.18 (0.19)	0.10 (0.09)	0.88 (0.21)	0.92 (0.11)	0.96 (0.07)	0.98 (0.04)
	Control	0.34 (0.11)	0.34 (0.09)	0.30 (0.20)	0.32 (0.16)	0.32 (0.27)	0.32 (0.13)

Degree of compliance for dietary recommendations after analyzing the food diaries. Values are given in mean with the standard deviations in parentheses. [0 = no compliance, 1 = consumption 100 % as recommended]

A regression analysis regarding the influence of the degree of compliance on the clinical parameters was performed for PI, GI, BOP, and PISA (Table 3). According to the regression analysis PI was significantly negatively associated with Omega-3 fatty acids and positively associated with fiber intake. GI was significantly negatively associated with Omega-3 fatty acid consumption and carbohydrate reduction. BOP und PISA were both significantly negatively associated with a reduction in carbohydrates.

**Table 3:** Regression analysis regarding clinical factors and degree of compliance

Clinical factor	Dietary factor	Coefficient	Standard deviation	*p*-value	95 % confidence interval
PI	Vitamin C	0.12	0.09	0.181	[-0.56; 0.30]
	Omega-3 FA	-0.26	0.11	0.022	[-0.49; -0.04]
	Fibers	0.33	0.15	0.021	[0.05; 0.63]
	Vitamin D	-0.10	0.83	0.815	[-0.18; 0.14]
	Antioxidants	-0.05	0.10	0.589	[-0,25; 0.14]
	Carbohydrate reduction	-0.11	0.13	0.375	[-0,38; 0.14]
GI	Vitamin C	-0.08	0.10	0.452	[-0.28; 0.12]
	Omega-3 FA	-0.42	0.12	0.001	[-0.69; -0.18]
	Fibers	0.01	0.16	0.952	[-0.31; 0.33]
	Vitamin D	0.15	0.09	0.089	[-0.02; 0.32]
	Antioxidants	-0.20	0.11	0.272	[-0.33; 0.09]
	Carbohydrate reduction	-0.59	0.15	0.001	[-0.88; -0.29]
BOP	Vitamin C	-0.09	0.11	0.394	[-0.30; 0.12]
	Omega-3 FA	-0.04	0.09	0.68	[-0.21; 0.14]
	Fibers	0.22	0.14	0.11	[-0.05; 0.49]
	Vitamin D	0.01	0.07	0.95	[-0.14; 0.15]
	Antioxidants	-0.16	0.09	0.06	[-0.33; 0.01]
	Carbohydrate reduction	-0.47	0.14	0.01	[-0.75; –0.29]
PISA	Vitamin C	-59.03	147.25	0.689	[-347.64; 229.58]
	Omega-3 FA	-229.65	136.18	0.092	[-496.43; 37.14]
	Fibers	239.73	200.92	0.233	[-154.07; 633.53]
	Vitamin D	113.11	113.65	0.320	[-109.64; 335.86]
	Antioxidants	-87.78	125.79	0.485	[-334.33; 158.76]
	Carbohydrate reduction	-581.59	197.88	0.003	[-969.42; -193.76]

Regression analysis regarding the influence of the degree of compliance on the clinical parameters with time as a categorical variable adjusted by age, gender and BMI. *Omega-3 FA* Omega-3 fatty acids, *PI* plaque index, *GI* gingival index, *BOP* bleeding on probing, *PISA* periodontal inflamed surface area

Intraclass Correlation was 0.91, showing a high level of reproducibility. The computed power of the study was 100 %.

## Discussion

The results of this pilot study showed that an oral health optimized diet can significantly reduce gingival and periodontal inflammation in a clinically important range without any changes in oral hygiene performance. The results of this study are in accordance with those of Baumgartner et al. [6], questioning the positive association of plaque and gingival inflammation in a changed dietary environment. Although the overall effects of this diet were impressive, it is hard to determine in retrospect which dietary element had the most impact on clinical parameters, even though the regression analysis showed significant associations of clinical factors primarily to carbohydrate-reduction and Omega-3 fatty acid intake. The control group showed a trend of increasing plaque values although this was not significant. This result was expectedly due to the absence of interdental hygiene during the study in individuals who prior to this performed interdental hygiene. But also irrespectively to the control group, the experimental group experienced a significant decrease in inflammatory parameters with regard to the baseline values, which is comparable with the Baumgartner et al. study.

In total, the results support the assumptions that a modern, Western lifestyle, including lots of refined carbohydrates and a high Omega-6 to Omega-3 fatty acid ratio promotes inflammatory processes [8]. As long as no specific dietary component can be made out for this decrease in periodontal inflammation, results have to probably be considered to be a mixture of inhibition and increased resolution of the inflammatory processes.

Regarding the intake of high-glycemic carbohydrates which promotes higher blood glucose levels and insulinemia, the results of this study seem to be in line with the known relationship of periodontal disease and diabetes [35] and the recently investigated periodontitis-associated genes ANRIL, VAMP3, and GLT6D1 which in some way seem to be related to glucose metabolism or glycosylation [36–38]. The association of carbohydrate consumption and gingivitis has been investigated in some earlier studies, also with impressive effects [7]. Looking into the literature there are several possible explanations for the inflammation triggering effect of carbohydrates. First of all, high glycemic index carbohydrates seem to directly promote inflammatory processes via NFkB-activation and oxidative stress [39, 40], and have been linked to higher C-reactive protein levels [41]. Secondly, high glycemic index carbohydrates may promote weight gain [42], with its associated inflammatory effects, based on an increase in adipokine secretion [43]. Looking at mean carbohydrate consumption in Germany, with more than 230 g per day or over 45 % of the total calorie intake [44], there may be associations with chronic inflammatory diseases in susceptible patients. Furthermore, the results are in line with Sidi & Ashley [45] who found a significant higher bleeding on probing in individuals on a high sugar diet compared to individuals on a low sugar diet, with no intergroup differences regarding the amount of plaque.

Regarding the effect of a decrease in the Omega-6 to Omega-3 fatty acid ratio, our results support the theory of resoleomics as stated by Serhan et al. [9], and the related periodontal studies [18, 19, 46]. Summarized, Serhan and colleagues discovered that the resolution of the inflammatory response is an active process based on metabolites of Omega-3 fatty acids, so called “specialized pro-resolving mediators” (SPM), rather than a passive event based on the elimination of the pro-inflammatory cytokines.

Looking at the intake of vitamins C and D and antioxidants, the results also support studies which have shown positive outcomes in this field. For a deeper discussion a reference should be made to Van der Velden et al. [11], where theoretical and clinical studies are described in detail.

The results raise several questions regarding the importance of dental plaque for the development of gingivitis/periodontitis and its impact on therapy. If dental examination reveals signs of an inflammation of the gingiva or the periodontium, representing a strongly host-mediated condition, a primary check of host-factors such as nutrition should be considered. The periodontist has the special opportunity – in contrast to that of other medical professions - to fairly easily get an impression about systemic inflammation, which could be a real contribution from periodontology in the prevention of other diseases related to chronic inflammation. This idea has been stated from by other authors as well [7, 47].

The primary limitations of this study can be seen in its small sample size, which is not a representative population, as well as with the rather uncontrolled intake of dietary components. However, to the best of the authors’ knowledge the study results are the currently only evidence beyond the Baumgartner et al. study in a randomized, controlled, interventional setting. Regarding the rather broad dietary recommendations, in the authors’ opinion it is very important to deliver a dietary protocol which is applicable to patients in daily practice. In other words, advising patients to implement this dietary pattern will show effects irrespective of whether they focus more on high glycemic index carbohydrate reduction, Omega-3 fatty acids, vitamin D, vitamin C, and/or antioxidants. Future studies should also include serum measurements of cytokines, cholesterol, HbA1c, vitamin D, etc. and also examine the oral microbiome. According to the exclusion criteria the study results are limited to patients with a mainly carbohydrate based diet. With regard to European epidemiological data, it can be assumed that this factor also applies to most individuals in the European population [48]. Furthermore, the assessment of dietary patterns using food diaries is not absolutely accurate. Thus, the presented data based on the food diaries can be seen as an indicator of compliance but not as a precise reflection of dietary patterns including quantitative values.

## Conclusion

Within the limitations, the presented dietary pattern including a diet low in carbohydrates, but rich in Omega-3 fatty acids, vitamins C and D, antioxidants and fiber significantly reduced periodontal inflammation in humans.

## Abbreviations

GI, gingival index; PI, plaque index; PD, pocket probing depths; BOP, bleeding on probing; PISA, periodontal inflamed surface area; BMI, body mass index; ICC, intraclass correlation coefficient.

## Additional files

Additional file 1: “S1 food diary.doc” contains the used food diary. (DOC 155 kb) Additional file 2: “Dataset.xlsx” contains the total anonymized dataset including group affiliation, gender, age, BMI, PI, GI, BOP, PISA, PD, CAL, and compliance values regarding the recommended nutrition. (XLSX 17 kb)

## References

[1] Kassebaum NJ, Bernabé E, Dahiya M, Bhandari B, Murray CJL, Marcenes W (2014). Global burden of severe periodontitis in 1990-2010 a systematic review and meta-regression. J Dent Res.

[2] Reich E, Hiller KA (1993). Reasons for tooth extraction in the western states of Germany. Community Dent Oral Epidemiol.

[3] Lang NP, Schätzle MA, Löe H (2009). Gingivitis as a risk factor in periodontal disease. J Clin Periodontol.

[4] Löe H, Theilade E, Jensen SB (1965). Experimental gingivitis in man. J Periodontol.

[5] Brecx MC, Fröhlicher I, Gehr P, Lang NP (1988). Stereological observations on long-term experimental gingivitis in man. J Clin Periodontol.

[6] Baumgartner S, Imfeld T, Schicht O, Rath C, Persson RE, Persson GR (2009). The impact of the stone age diet on gingival conditions in the absence of oral hygiene. J Periodontol.

[7] Hujoel P (2009). Dietary carbohydrates and dental-systemic diseases. J Dent Res.

[8] Bosma-den Boer MM, van Wetten M-L, Pruimboom L (2012). Chronic inflammatory diseases are stimulated by current lifestyle: how diet, stress levels and medication prevent our body from recovering. Nutr Metab.

[9] Serhan CN, Chiang N, Dalli J (2015). The resolution code of acute inflammation: Novel pro-resolving lipid mediators in resolution. Semin Immunol.

[10] Simopoulos AP (2006). Evolutionary aspects of diet, the omega-6/omega-3 ratio and genetic variation: nutritional implications for chronic diseases. Biomed Pharm.

[11] Van der Velden U, Kuzmanova D, Chapple ILC (2011). Micronutritional approaches to periodontal therapy. J Clin Periodontol.

[12] Chapple ILC, Milward MR, Ling-Mountford N, Weston P, Carter K, Askey K (2012). Adjunctive daily supplementation with encapsulated fruit, vegetable and berry juice powder concentrates and clinical periodontal outcomes: a double-blind RCT. J Clin Periodontol.

[13] Merchant AT, Pitiphat W, Franz M, Joshipura KJ (2006). Whole-grain and fiber intakes and periodontitis risk in men. Am J Clin Nutr.

[14] Adler CJ, Dobney K, Weyrich LS, Kaidonis J, Walker AW, Haak W (2013). Sequencing ancient calcified dental plaque shows changes in oral microbiota with dietary shifts of the Neolithic and Industrial revolutions. Nat Genet.

[15] Kim HS, Park JW, Yeo SI, Choi BJ, Suh JY (2006). Effects of high glucose on cellular activity of periodontal ligament cells in vitro. Diabetes Res Clin Pract.

[16] Liu J, Jiang Y, Mao J, Gu B, Liu H, Fang B (2013). High levels of glucose induces a dose-dependent apoptosis in human periodontal ligament fibroblasts by activating caspase-3 signaling pathway. Appl Biochem Biotechnol.

[17] Mustafa M, Zarrough A, Bolstad AI, Lygre H, Mustafa K, Hasturk H (2013). Resolvin D1 protects periodontal ligament. Am J Physiol Cell Physiol.

[18] El-Sharkawy H, Aboelsaad N, Eliwa M, Darweesh M, Alshahat M, Kantarci A (2010). Adjunctive treatment of chronic periodontitis with daily dietary supplementation with omega-3 Fatty acids and low-dose aspirin. J Periodontol.

[19] Elkhouli AM (2011). The efficacy of host response modulation therapy (omega-3 plus low-dose aspirin) as an adjunctive treatment of chronic periodontitis (clinical and biochemical study). J Periodont Res.

[20] Jimenez M, Giovannucci E, Krall Kaye E, Joshipura KJ, Dietrich T. Predicted vitamin D status and incidence of tooth loss and periodontitis. Public Health Nutr. 2013;50:666-73. 10.1017/S1368980013000177 PMC4911807 23469936

[21] Nebel D, Svensson D, Arosenius K, Larsson E, Jönsson D, Nilsson B-O. 1α,25-dihydroxyvitamin D3 promotes osteogenic activity and downregulates proinflammatory cytokine expression in human periodontal ligament cells. J Periodont Res. 2014;34:299-304. 10.1111/jre.12249 25495336

[22] Amaliya, Timmerman MF, Abbas F, Loos BG, Van der Weijden GA, Van Winkelhoff AJ, et al. Java project on periodontal diseases: the relationship between vitamin C and the severity of periodontitis. J Clin Periodontol. 2007;34:299–304. 10.1111/j.1600-051X.2007.01053.x 17378886

[23] Merchant AT (2008). Plasma vitamin C is inversely associated with periodontitis. J Evid Based Dent Pract.

[24] Nishida M, Grossi SG, Dunford RG, Ho AW, Trevisan M, Genco RJ (2000). Dietary vitamin C and the risk for periodontal disease. J Periodontol.

[25] Yan Y, Zeng W, Song S, Zhang F, He W, Liang W (2013). Vitamin C induces periodontal ligament progenitor cell differentiation via activation of ERK pathway mediated by PELP1. Protein Cell.

[26] Muniz FWMG, Nogueira SB, Mendes FLV, Rösing CK, Moreira MMSM, de Andrade GM (2015). The impact of antioxidant agents complimentary to periodontal therapy on oxidative stress and periodontal outcomes: A systematic review. Arch Oral Biol.

[27] Nizam N, Discioglu F, Saygun I, Bal V, Avcu F, Ozkan CK (2014). The effect of α-tocopherol and selenium on human gingival fibroblasts and periodontal ligament fibroblasts in vitro. J Periodontol.

[28] Chapple ILC, Milward MR, Dietrich T (2007). The prevalence of inflammatory periodontitis is negatively associated with serum antioxidant concentrations. J Nutr.

[29] Feinman RD, Pogozelski WK, Astrup A, Bernstein RK, Fine EJ, Westman EC (2015). Dietary carbohydrate restriction as the first approach in diabetes management: critical review and evidence base. Nutrition.

[30] Löe H, Silness J (1963). Periodontal Disease in pregnancy. I. Prevalence and severity. Acta Odontol Scand.

[31] van Woudenbergh GJ, Theofylaktopoulou D, Kuijsten A, Ferreira I, van Greevenbroek MM, van der Kallen CJ (2013). Adapted dietary inflammatory index and its association with a summary score for low-grade inflammation and markers of glucose metabolism: the Cohort study on Diabetes and Atherosclerosis Maastricht (CODAM) and the Hoorn study. Am J Clin Nutr.

[32] Silness J, Löe H (1964). Periodontal Disease in pregnancy. II. Correlation between oral hygiene and periodontal condition. Acta. Odontol Scand.

[33] Nesse W, Abbas F, van der Ploeg I, Spijkervet FKL, Dijkstra PU, Vissink A (2008). Periodontal inflamed surface area: quantifying inflammatory burden. J Clin Periodontol.

[34] AAP (2015). American Academy of Periodontology Task Force report on the update to the 1999 classification of periodontal diseases and conditions. J Periodontol.

[35] Chapple ILC, Genco R, Working group 2 of joint EFP/AAP workshop (2013). Diabetes and periodontal diseases: consensus report of the Joint EFP/AAP Workshop on Periodontitis and Systemic Diseases. J Clin Periodontol.

[36] Schaefer AS, Richter GM, Nothnagel M, Manke T, Dommisch H, Jacobs G (2010). A genome-wide association study identifies GLT6D1 as a susceptibility locus for periodontitis. Hum Mol Genet.

[37] Schaefer AS, Bochenek G, Manke T, Nothnagel M, Graetz C, Thien A (2013). Validation of reported genetic risk factors for periodontitis in a large-scale replication study. J Clin Periodontol.

[38] Schaefer AS, Bochenek G, Jochens A, Ellinghaus D, Dommisch H, Güzeldemir-Akçakanat E (2015). Genetic evidence for PLASMINOGEN as a shared genetic risk factor of coronary artery disease and periodontitis. Circ Cardiovasc Genet.

[39] Dickinson S, Hancock DP, Petocz P, Ceriello A, Brand-Miller J (2008). High-glycemic index carbohydrate increases nuclear factor-kappaB activation in mononuclear cells of young, lean healthy subjects. Am J Clin Nutr.

[40] Hu Y, Block G, Norkus EP, Morrow JD, Dietrich M, Hudes M (2006). Relations of glycemic index and glycemic load with plasma oxidative stress markers. Am J Clin Nutr.

[41] Liu S, Manson JE, Buring JE, Stampfer MJ, Willett WC, Ridker PM (2002). Relation between a diet with a high glycemic load and plasma concentrations of high-sensitivity C-reactive protein in middle-aged women. Am J Clin Nutr.

[42] Brand-Miller JC, Holt SHA, Pawlak DB, McMillan J (2002). Glycemic index and obesity. Am J Clin Nutr.

[43] Piya MK, McTernan PG, Kumar S (2013). Adipokine inflammation and insulin resistance: the role of glucose, lipids and endotoxin. J Endocrinol.

[44] Hauner H, Bechthold A, Boeing H, Brönstrup A, Buyken A, Leschik-Bonnet E (2012). Evidence-based guideline of the German Nutrition Society: carbohydrate intake and prevention of nutrition-related diseases. Ann Nutr Metab.

[45] Sidi AD, Ashley FP (1984). Influence of frequent sugar intakes on experimental gingivitis. J Periodontol.

[46] Hasturk H, Kantarci A, Van Dyke TE (2012). Paradigm shift in the pharmacological management of periodontal diseases. Front Oral Biol.

[47] Genco RJ, Genco FD (2014). Common risk factors in the management of periodontal and associated systemic diseases: the dental setting and interprofessional collaboration. J Evid Based Dent Pract.

[48] Elmadfa I, Meyer A, Nowak V, Hasenegger V, Putz P, Verstraeten R (2009). European Nutrition and Health Report 2009. Ann Nutr Metab.

