# Erratum to: An oral health optimized diet can reduce gingival and periodontal inflammation in humans - a randomized controlled pilot study

**DOI:** 10.1186/s12903-016-0304-y

**Published:** 2016-10-06

**Authors:** J. P. Woelber, K. Bremer, K. Vach, D. König, E. Hellwig, P. Ratka-Krüger, A. Al-Ahmad, C. Tennert

**Affiliations:** 1 Department of Operative Dentistry and Periodontology, Center for Dental Medicine, Medical Center – University of Freiburg, Hugstetter Str. 55, Freiburg, Germany; 2 Department of Medical Biometry and Statistics, Medical Center – University of Freiburg, Freiburg, Germany; 3 Institute of Sports and Sports Science, Medical Center – University of Freiburg, Freiburg, Germany

## Erratum

Unfortunately, the original version of this article [1] was missing funding information within the acknowledgements section.

The article processing charge was funded by the German Research Foundation (DFG) and the Albert Ludwigs University Freiburg in the funding programme Open Access Publishing.
