# Supplementary material for: An oral health optimized diet can reduce gingival and periodontal inflammation in humans - a randomized controlled pilot study
Source: BMC Oral Health. 2016 Jul 26;17:28. doi: 10.1186/s12903-016-0257-1 (PMC4962497; doi:10.1186/s12903-016-0257-1)
Supplement: Additional file 1: — “S1 food diary.doc” contains the used food diary. (DOC 155 kb) [file 12903_2016_257_MOESM1_ESM.doc]

**Food diary**

Date:

Weight:

| Time | Kind of food and amount |
| --- | --- |
|  |  |
|  |  |
|  |  |
|  |  |
|  |  |
|  |  |
|  |  |
|  |  |
|  |  |
|  |  |
|  |  |
|  |  |
